# Recent Advances in Fluorescent Probes for Imaging Enzyme Activity in NETosis

**DOI:** 10.1002/cbic.70483

**Published:** 2026-07-30

**Authors:** Enebie Ramos Cáceres, Marijn J. Hollander, Kimberly M. Bonger

**Affiliations:** ^1^ Chemical Biology & Immunology Leiden Institute of Chemistry Leiden University Leiden The Netherlands; ^2^ Department of Synthetic Organic Chemistry Institute for Molecules and Materials Radboud University Nijmegen The Netherlands

**Keywords:** biosensors, enzymes, fluorescent probes, NETosis, neutrophil

## Abstract

Neutrophil extracellular traps (NETs) are web‐like structures of DNA released by neutrophils during a specialized form of cell death called NETosis. Originally described as a mechanism of pathogen neutralization, NETs have since been implicated in a growing number of pathological conditions, including inflammatory diseases, autoimmunity, cancer, and neurodegeneration. The complexity and rapid dynamics of neutrophils and NET formation pose significant challenges for conventional approaches, highlighting a need for tools that can report molecular events with high spatial and temporal precision. Fluorescent probes have proven invaluable in this regard, offering both the sensitivity and selectivity required to study this process. This review provides a comprehensive overview of recent progress on the development of fluorescent probes for imaging NETosis, focusing on their design principles, biological applications, and key limitations. We discuss how these tools have advanced our understanding of the stimulus‐dependent and spatiotemporally distinct activation dynamics of enzymes central to NETosis. We further highlight remaining challenges and outline design strategies that we believe will guide the development of next‐generation tools to investigate neutrophil biology in health and disease.

## Graphical abstract

Fluorescent probes enable real‐time visualization of neutrophil effector functions. This review summarizes current probe design strategies and emerging tools for imaging key enzymatic activities, with emphasis on mechanisms underlying NETosis, a lytic form of neutrophil extracellular trap (NET) formation. We discuss current limitations, challenges, and future directions in the field.

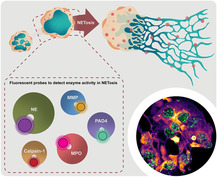

## 1 Introduction

Neutrophils are the most abundant circulating leukocyte and serve as one of the first lines of defense against invading pathogens and acute inflammation [1]. Morphologically defined by their multilobulated nuclei, neutrophils possess a versatile arsenal to neutralize their targets, including degranulation (i.e., the release of cytotoxic molecules from cytoplasmic granules), phagocytosis, and neutrophil extracellular trap (NET) formation [1, 2]. Among these, lytic NET formation, commonly termed NETosis, has emerged over the past two decades as a particularly complex and dynamic effector function. NETosis proceeds through tightly regulated signaling cascades and enzymatic activities that culminate in chromatin decondensation and the expulsion of DNA coated with antimicrobial proteins [1, 2]. Although initially studied in the context of the innate immune response against pathogens, NET formation can also be triggered by exogenous and endogenous stimuli such as small molecules [3], cytokines [4], autoantigens [5], and autoantibodies [5]. Additionally, NETs have been shown to activate other immune cells and affect the adaptive immune response [6].

While NETs are typically beneficial during infection, dysregulated or excessive NET formation can cause damage to the host. NETs can act as a source of autoantigens and release an overabundance of cytotoxic proteins that may cause cell death and tissue damage. As such, neutrophils and NETs play a significant role in several pathological processes that result in chronic inflammation [7], autoimmune disorders [8], thrombosis [9], cancer [10], and neurodegeneration [11]. The molecular pathways that govern neutrophil activation and NET formation are highly context‐dependent and depend on the nature of the stimuli, the cellular environment, and cross‐talk with other immune signals [1, 2, 12]. However, the precise roles and functional significance of the enzymes driving NET formation and release remain incompletely understood and, in certain cases, controversial.

To address these uncertainties, analytical tools capable of monitoring enzymatic activities in living cells with both high spatial and temporal resolution are highly desired. Traditional methods, such as immunofluorescence microscopy, rely on fixed samples, which can disrupt structural cellular elements and fail to fully capture the complexity and highly dynamic nature of processes such as NETosis [13]. As such, molecular probes have helped overcome these limitations by enabling minimally perturbative, real‐time monitoring of biological processes in live cells [14, 15]. Specifically, advances in probe design over the past two decades have established fluorescent probes as central tools due to their sensitivity, versatility, and ease of use [14, 15].

In this review, we first summarize the molecular mechanisms leading to NETosis and highlight key enzymatic players in the process. We then provide a comprehensive overview of recent progress in the development of fluorescent probes for imaging these targets. Throughout, we discuss fluorescent probe design principles, biological applications, and limitations of each probe class, with emphasis on research from the past 5 years. We conclude by addressing remaining challenges in the field and provide a perspective on the design strategies that we believe will guide the development of next‐generation fluorescent probes to study neutrophil biology in health and disease.

## 2 Lytic NET Formation: NETosis

**FIGURE 1:**
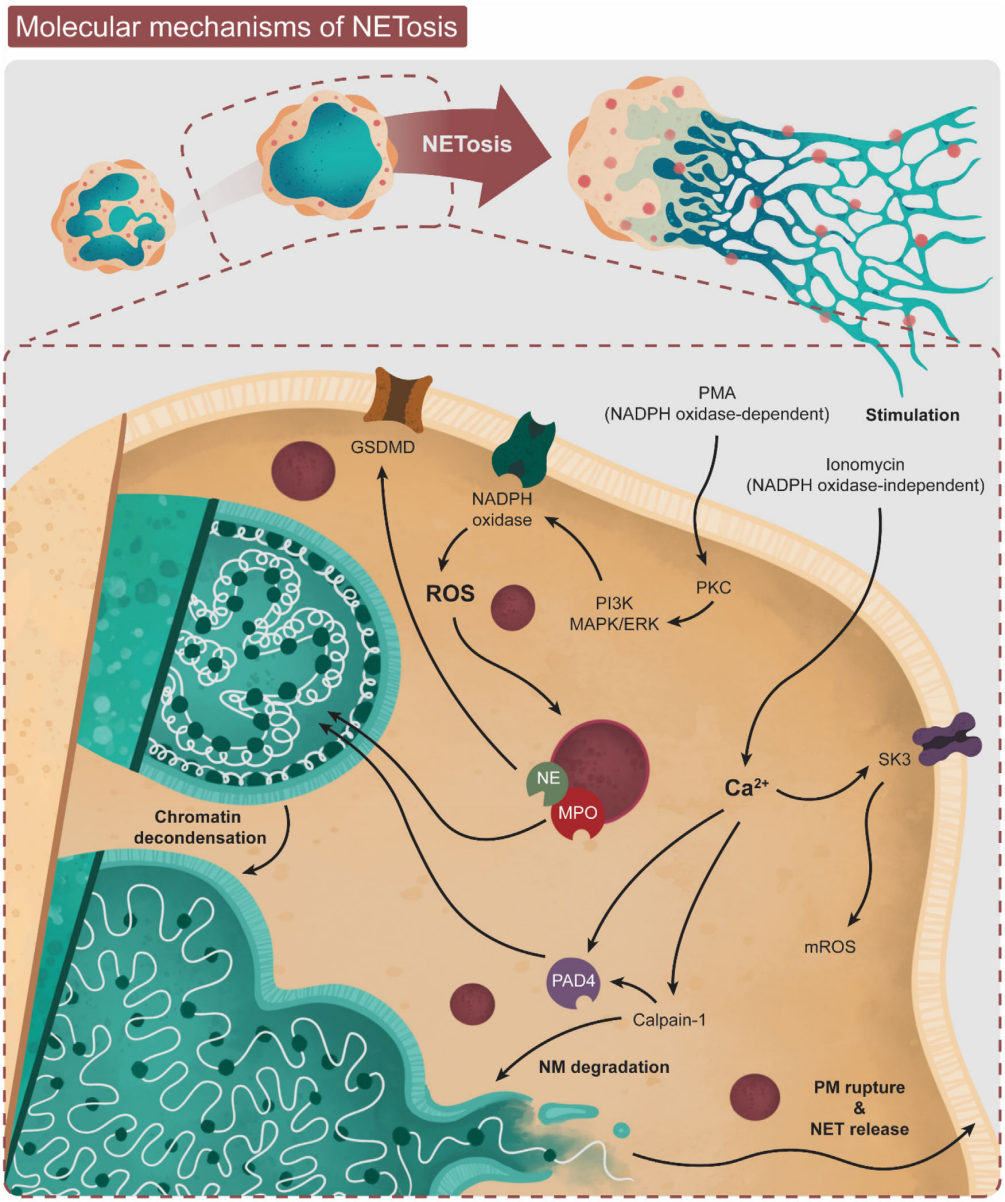
Molecular mechanisms of NADPH oxidase‐dependent and ‐independent NETosis. In the NADPH oxidase‐dependent pathway, PMA stimulation leads to the assembly of the NADPH oxidase complex through PKC, PI3K, and MAPK/ERK signaling. NADPH oxidase generates ROS, releasing MPO and NE from cytoplasmic granules. NE activates GSDMD and translocates to the nucleus together with MPO, where they aid in chromatin decondensation. In the NADPH oxidase‐independent pathway, ionomycin stimulation results in a sudden increase in intracellular Ca^2+^ concentration, which activates SK3 and leads to the production of mROS. PAD4 and calpain‐1 also become active. PAD4 citrullinates histone tails, aiding in chromatin decondensation, while calpain‐1 weakens the nuclear membrane (NM). In both pathways, the molecular processes ultimately lead to plasma membrane (PM) rupture and the release of NETs. Parts of this figure are reprinted (adapted) from ref. [16]. and with permission from ref. [17]. Copyright 2026 American Chemical Society.

Based on their induction mechanisms and regulatory features, NETosis pathways can be broadly classified into two major types: NADPH oxidase‐dependent and NADPH oxidase‐independent (Figure 1). The NADPH oxidase‐dependent pathway is the more extensively studied and characterized, with most mechanistic insights derived from studies using bacteria or biochemical stimuli, such as phorbol 12‐myristate 13‐acetate (PMA) and bacterial lipopolysaccharides (LPS). PMA is a synthetic activator of the protein kinase C (PKC) family of enzymes, which initiates signaling through the phosphoinositide 3‐kinase (PI3K) and mitogen‐activated protein kinase/extracellular signal‐regulated kinase (MAPK/ERK) signaling cascades [18]. LPS, by contrast, has been shown to activate the Toll‐like receptor 4 (TLR4) on the neutrophil surface and proceed through c‐Jun N‐terminal kinases (JNK) [19]. In both cases, signaling converges in the assembly of the NADPH oxidase complex and the generation of reactive oxygen species (ROS).

The production of ROS through the NADPH oxidase‐dependent pathway has been reported to be essential for the release of myeloperoxidase (MPO) and neutrophil elastase (NE) [20]. MPO is a heme peroxidase with strong antimicrobial properties and uses H_2_O_2_ to generate hypohalous acids such as HOCl, which aid in pathogen neutralization by neutrophil effector functions [20]. NE is a serine protease with microbicidal activity and can break down host tissue [20]. They both localize to the azurosome, a protein complex associated with the membranes of neutrophil primary granules [20]. MPO‐mediated oxidative reactions promote the dissociation of NE, enabling its release into the cytosol [20]. This licenses NE to degrade actin filaments and translocate to the nucleus, where it proteolyzes histone H3 tails and contributes to chromatin decondensation [20, 21]. As the nuclear envelope is degraded, decondensed chromatin mixes with the cytoplasm, and eventual rupture of the plasma membrane releases NETs. Additionally, NE cleaves gasdermin D (GSDMD) to its active form, which can form pores in both the plasma and granule membranes, thereby facilitating NET release [22].

The NADPH oxidase‐independent pathway, in contrast, is heavily dependent on Ca^2+^ fluctuations and is typically induced by calcium ionophores such as A23187 and ionomycin. These molecules trigger a rapid rise in intracellular Ca^2+^ concentration, activating the small conductance calcium‐activated potassium channel 3 (SK3) and promoting the production of mitochondrial ROS (mROS) [23]. High intracellular Ca^2+^ concentrations also activate protein arginine deiminase 4 (PAD4), a Ca^2+^‐dependent hydrolase highly expressed in neutrophils [24, 25]. PAD4 catalyzes the citrullination of arginine residues on histone tails, promoting nuclear decondensation by removing the positive charges that help maintain the electrostatic interaction between histones and DNA [24, 25]. It has been found that PAD4 acts in concert with calpain‐1, a Ca^2+^‐dependent protease, to weaken the nuclear envelope, thereby promoting chromatin decondensation and NET release [26, 27].

## 3 Principles of Fluorescent Probe Design

Fluorescent probes for detecting enzymatic activity typically incorporate a recognition element based on known substrates, ligands, reversible inhibitors, or covalent inhibitors [28, 29]. Those based on known substrates are often designed around specific enzymatic cleavage events or modifications. Fluorescent probes that utilize ligands or reversible inhibitors rely on affinity‐based interactions with the enzyme. Probes employing covalent inhibitors, on the other hand, can covalently modify the active site of the target enzyme. The last type is commonly known as activity‐based probes (ABPs), a concept originally developed by Cravatt and Bogyo for fluorescent probes incorporating electrophilic traps against proteases [30, 31]. Today, ABPs generally describe probes using covalent inhibitors that exploit the natural catalytic cycle of the enzyme, forming adducts only after undergoing enzyme‐mediated transformation. Unlike most substrate‐based probes, which benefit from signal amplification as a single enzyme can process multiple substrates, ABPs irreversibly inactivate their targets [32]. However, this covalent mechanism enables precise visualization and localization of active enzymes within complex biological systems [32].

**FIGURE 2:**
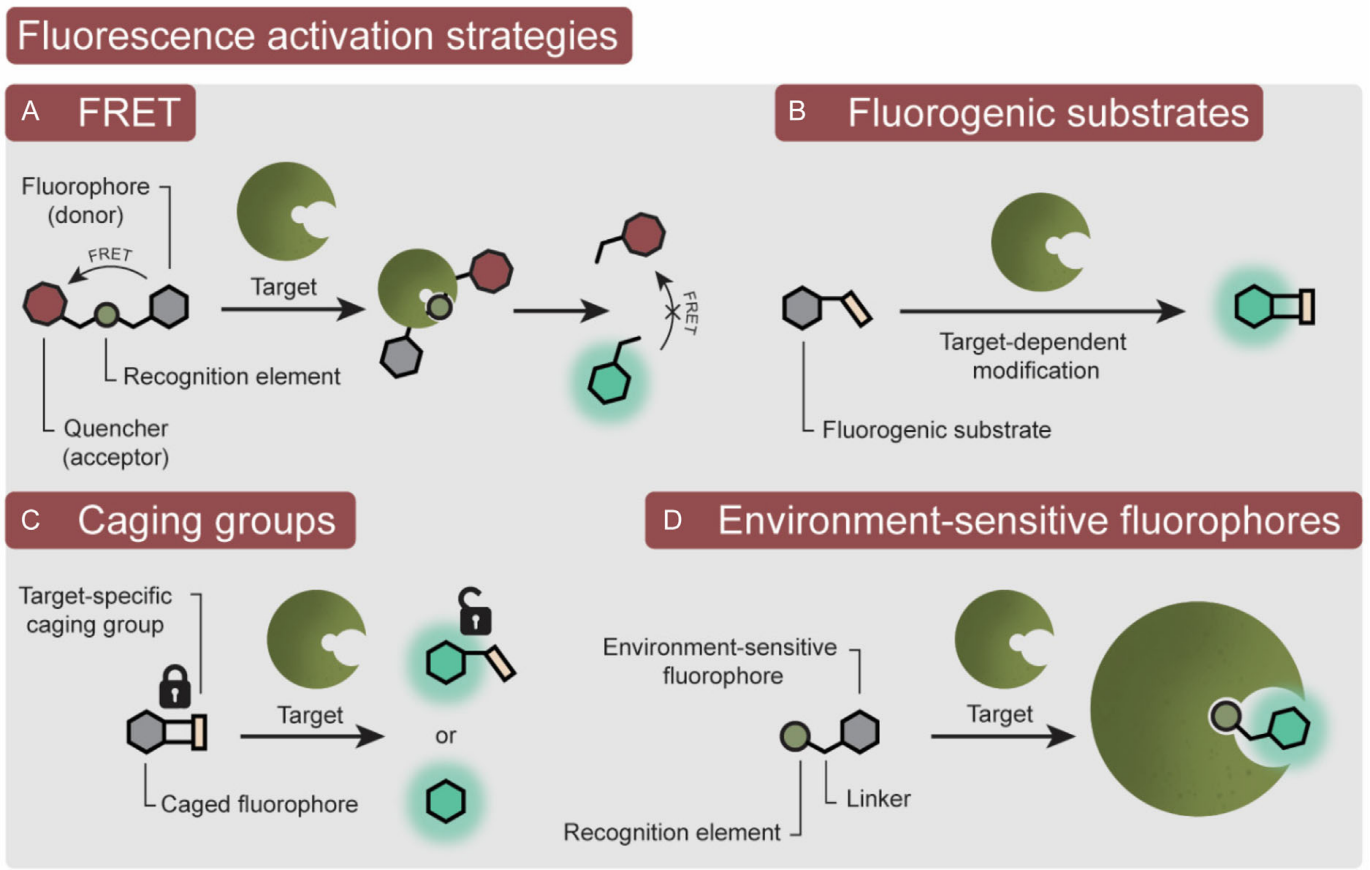
Types of fluorescence activation strategies. (A) FRET‐based probes employ a donor and an acceptor, leading to fluorescence quenching or ratiometric probes. (B) Fluorogenic substrates that undergo target‐dependent modifications to become fluorescent. (C) Application of target‐specific caging groups that can be modified or removed to induce fluorescence. (D) Employing fluorophores sensitive to environmental changes (e.g., pH, redox state, hydrophobicity).

Fluorescent probes used for the visualization of biological processes in their simplest form only require a constitutively fluorescent reporter. However, the incorporation of fluorescence activation strategies can substantially increase signal quality by improving contrast and enabling temporal and spatial control (Figure 2A–D). One commonly used example is the disruption of Förster resonance energy transfer (FRET) [15, 33]. In FRET‐based probes, fluorescence is quenched when a donor fluorophore transfers energy to a nearby acceptor fluorophore or quencher (Figure 2A). Cleavage of the acceptor, typically through a linker targeted by the enzyme of interest, ends this interaction and restores donor fluorescence. If the donor and acceptor are a pair of fluorophores, the ratio between their fluorescence emission is affected by interaction with the target, giving rise to ratiometric probes [15, 34]. If the donor is a fluorophore and the acceptor is a quencher, this strategy is commonly applied to ABP designs to create quenched ABPs (qABPs) [28]. Fluorescence activation can also be achieved through the incorporation of fluorogenic substrates, where a chemical compound undergoes a physicochemical, photochemical, or chemoenzymatic modification to become fluorescent (Figure 2B) [15, 35, 36, 37]. Variations of fluorogenic strategies include incorporating a caging group that can be enzymatically cleaved or modified (Figure 2C) and the use of environment‐sensitive fluorophores (Figure 2D). Environment‐sensitive fluorophores exhibit increased fluorescence in specific environments, such as compartments with high or low pH levels, oxidizing conditions, or within hydrophobic structures like lipid droplets, membranes, or the surface of enzymes [37].

Fluorescent probe performance in cellular systems is strongly influenced by factors such as live‐cell compatibility, molecular size, plasma membrane permeability, and specificity [15, 38]. For instance, substrate‐based probes are often well‐suited for live‐cell studies because they are generally small and cell‐permeable [15]. However, they can suffer from limited specificity due to signal diffusion and overlap in substrate preferences within enzyme families [15]. ABPs provide specific labeling of active enzymes, but the nature of the electrophilic trap can be a source of background fluorescence due to off‐target effects [39]. Fluorescence activation strategies also vary in live‐cell applicability: FRET‐based fluorescent probes can be used directly in living cells, but their size often affects plasma membrane permeability [38, 40], whereas environment‐sensitive fluorophores or caged substrates may be influenced by subcellular localization, probe polarity, or stability [37, 41]. Taken together, the choice of probe design involves balancing sensitivity, selectivity, and imaging context, with considerations of probe size, fluorescence activation strategy, and the spatial distribution of the target enzyme being crucial for successful implementation.

While fluorescent probes have become indispensable tools to study immune cells [42], neutrophils still present unique imaging challenges due to their dynamic phenotypes, rapid responses, and short lifespans [1, 2]. Additionally, they are particularly susceptible to environmental stress, and the kinetics of the molecular mechanisms behind their effector functions, especially NETosis, far outpace traditional immunostaining methods and conventional clickable probes [43]. To overcome these limitations, fluorescent probes have been successfully developed to study neutrophil activity in real time [42]. In the following sections, we outline general strategies employed to target key enzymes involved in NETosis and highlight recent advances, providing examples of how these probes have been used to advance our knowledge of neutrophil effector functions.

## 4 Fluorescent Probes to Image Enzyme Activity in NETosis

Most fluorescent probes for studying enzyme activity in NETosis are designed to detect protease activity, with the most common targets being neutrophil serine proteases (NSPs). NSPs are stored in neutrophil granules and are involved in both intracellular and extracellular pathogen breakdown. Members of the group include NE, cathepsin G (CatG), proteinase 3 (PR3), and neutrophil serine protease 4 (NSP4) [44, 45]. Other proteases include matrix metalloproteinases (MMPs) and, more recently, calpain‐1. Fluorescent probes for these enzymes have been successfully employed to study neutrophil functions, but their recognition elements often rely on peptide sequences. As such, they exhibit poor plasma membrane permeability, and achieving selectivity for specific enzymes can be challenging due to structural similarities and overlapping substrate specificities [46, 47, 48]. Nevertheless, the well‐defined substrate preference of these proteases has made them the most extensively targeted enzyme class in the field.

By comparison, developing fluorescent probes for nonproteolytic enzymes involved in NETosis is often more challenging. Key enzymes such as MPO and PAD4 are less suitable for conventional substrate‐based probe designs, as the former produces hypohalous acids from H_2_O_2_ and the latter catalyzes the conversion of arginine to citrulline, a post‐translational modification. In both cases, fluorescent probe designs that directly target the enzyme are limited by the availability of selective small‐molecule ligands or inhibitors. Consequently, the activity of these enzymes is frequently assessed indirectly through their enzymatic products. However, this approach comes with its own challenges, including enzymatic product or readout diffusion, and a lack of spatial resolution.

### 4.1 NE and Other NSPs

NE is one of the most well‐studied NSPs and has a broad substrate scope, from extracellular matrix (ECM) proteins such as elastin, fibronectin, and collagen, to cytokines [49]. Jugniot et al. reviewed strategies and examples of fluorescent probes for imaging NE activity, with a focus on peptide‐based probes [50]. Since then, several novel probes targeting NE have been reported.

**FIGURE 3:**
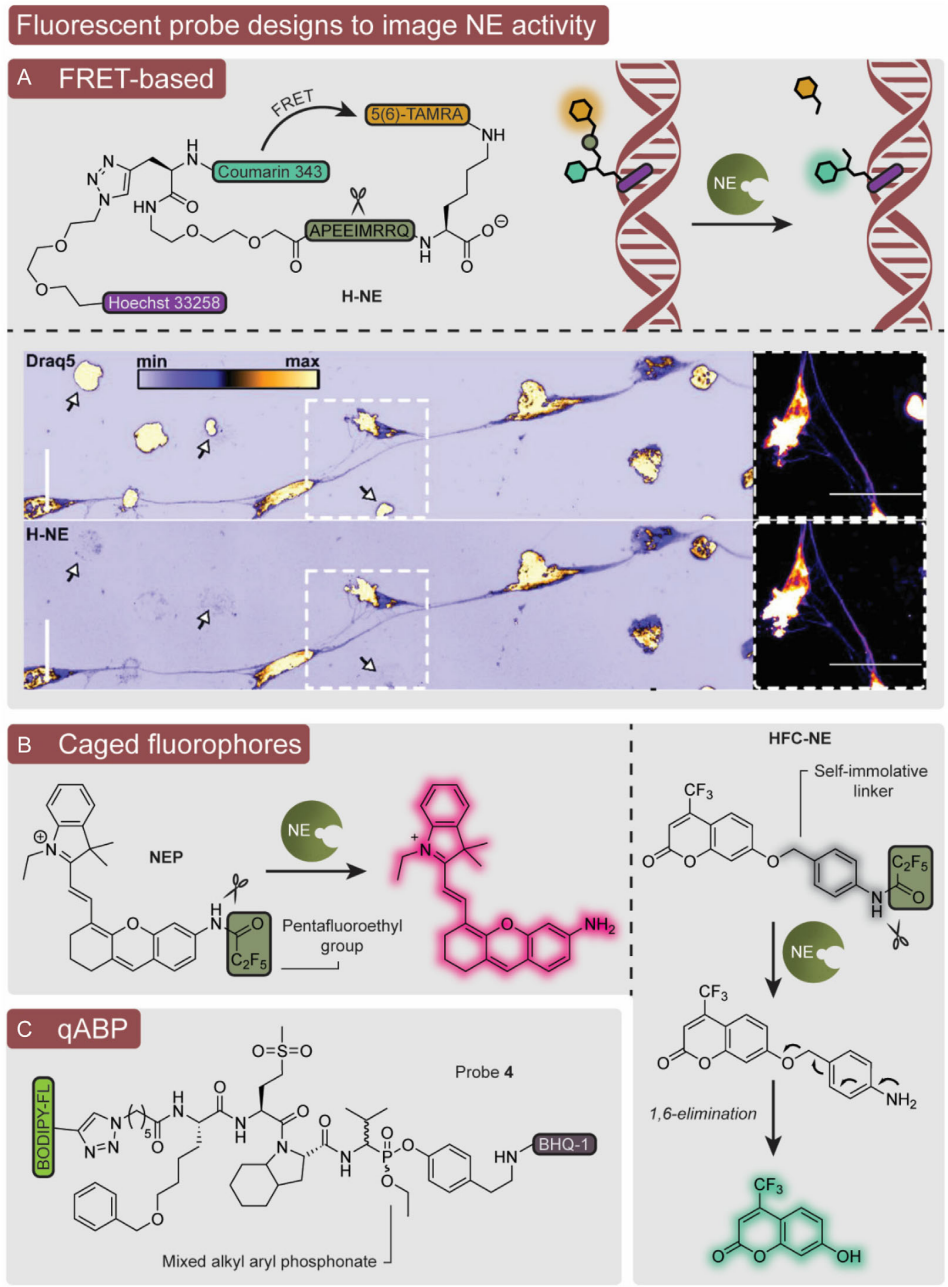
Overview of fluorescent probe designs to image NE activity. (A) Design of FRET‐based approaches (top) exemplified by **H‐NE** [51]. Confocal tilescan of fixed neutrophils after PMA stimulation and incubated with Draq5 and **H‐NE** (bottom). Scale bars = 30 µm. Reprinted (adapted) with permission from ref. [51]. Copyright 2020 American Chemical Society. (B) Caged fluorophore strategies featuring the NE‐specific pentafluoroethyl group. (C) qABP designs exemplified by probe **4**.

Fluorescent probes based on known substrates featuring FRET approaches are commonly employed to visualize the activity of proteases, including NE (Figure 3A, top). To image NE activity specifically associated with NETs of human neutrophils, the Schultz group reported a ratiometric probe (**H‐NE**) containing an NE‐specific peptide (APEEIMRRQ) conjugated to a pair of FRET sensors and a derivative of Hoechst 33258, a DNA‐binding dye [51, 52]. The design is based on their previously reported NEmo sensors [52], featuring coumarin 343 and 5(6)‐TAMRA as the donor and acceptor, respectively. Hoechst 33258 served as a targeting moiety to bind the probe to the minor groove of DNA and detect DNA‐associated NE activity in NETs from neutrophils stimulated by PMA [51]. Using a commercial DNA stain (Draq5), they showed an almost complete overlap of the **H‐NE** signal with NETs, and used the probe in live‐cell imaging studies to detect DNA‐associated NE activity after nuclear envelope rupture and NETosis (Figure 3A, bottom). In addition, they showed that while **H‐NE** did not penetrate live cells, it did enter the nuclei of permeabilized neutrophils, owing to the high molecular size and cell impermeability typical of FRET probes. **H‐NE** was also used to image NE activity in the sputum of cystic fibrosis patients and ex vivo mouse lung slices.

To image total NE activity, Mavileti et al. developed self‐quenching probes featuring the NE‐specific tripeptide APA connected to SQ‐215 and SQ‐46, a FRET pair of squaraine dyes [53]. They used these probes to image NE activity in monosodium urate crystal‐induced NETs in mice. To achieve higher signal‐to‐background ratios, the Bradley group used a multivalent scaffold consisting of three identical NE‐cleavable peptides (EEINleRR) to develop tribranched, deeply quenched probes [54, 55]. Early designs conjugated each peptide to 5‐carboxyfluorescein (5‐FAM) and methyl red (MR) as a quencher [54]. Later, they employed three cyanine 5 (Cy5) fluorophores and one central QSY21 quencher [55]. The probes displayed high selectivity for NE over other NSPs and enabled specific visualization of NE in NETosis induced by PMA or by neutrophil activation with *N*‐formylmethionyl‐leucyl‐phenylalanine (fMLP), a potent chemotactic factor. The multivalent structure is a notable design advance, as it improves quenching efficiency and signal amplification simultaneously. However, the increased molecular size and complexity further restrict cell permeability compared to simpler FRET probes.

Another common design for NE probes involves using caged fluorophores (Figure 3B). The Yang group synthesized the first nonpeptide‐based fluorogenic probe for NE, which featured the pentafluoroethyl group as an NE‐specific recognition element and 7‐amino‐4‐trifluoromethylcoumarin as the caged fluorophore [56]. Later, it was developed into a more selective and sensitive probe by employing a hemicyanine‐based caged fluorophore (**NEP**) [57]. The probe was used to visualize NE trafficking, uptake, and upregulation in cell lines, as well as NE activity in mouse models of acute lung injury. Since then, different variations of the original design have been developed, primarily focused on the nature of the caged fluorophore [58, 59, 60, 61, 62]. In addition, several lines of research have employed self‐immolative linkers to expand the chemical repertoire of fluorophores compatible with the pentafluoroethyl group [63, 64, 65, 66]. For instance, Li et al. conjugated 4‐trifluoromethyl‐7‐hydroxyl coumarin to the pentafluoroethyl group through a *p‐*aminobenzyl alcohol linker to create a fluorogenic probe (**HFC‐NE**) [63]. Upon cleavage of the recognition moiety by NE, the self‐immolative linker undergoes 1,6‐elimination to release the fluorophore. This strategy was used to image NE activity in cell lines and zebrafish, but not in human neutrophils.

ABPs have also been reported for NE, with many employing phosphonate esters as electrophilic traps (Figure 3C) [32, 67, 68]. The Drag group employed a hybrid combinatorial substrate library (HyCoSuL) profiling approach to obtain the optimal substrate for NE [69]. This strategy allowed them to scan the S1 to S4 specificity pockets of NE to find preferred sequences with natural or unnatural amino acids at the P1 to P4 positions. This peptide substrate was then converted into an ABP using diphenyl phosphonate as an electrophilic trap and biotin or sulfo‐Cy5 as a reporter moiety and employed to visualize NE activity in NETosis triggered by PMA stimulation in neutrophils [69, 70]. Importantly, phosphonate esters are poor leaving groups for qABP designs. Therefore, the Verhelst group employed both mixed alkyl aryl phosphonates and phosphinate esters to design qABPs towards NE [71, 72, 73, 74, 75]. In their most recent work, Kahler et al. employed the NE peptide substrate found by the Drag group with a valine at P1. They incorporated a mixed alkyl aryl phosphonate electrophilic trap, a BODIPY‐FL fluorophore, and BHQ‐2 as a quencher to create a probe that could be used in live cells (probe **4**) [75]. Indeed, they employed the probe to image NSP activity in neutrophils stimulated with PMA in real time. However, as with FRET‐based probes, the synthesized qABPs remain largely cell‐impermeable in unstimulated cells. This underscores the need for intracellular probes that do not require membrane disruption to study NE activity and early enzymatic events in NETosis.

The remaining members of the NSPs, CatG, PR3, and NSP4, have a much more modest repertoire of fluorescent probes. Their differences in substrate specificity have been explored using HyCoSuL to find selective ABPs, and probes have been made and simultaneously used to visualize NSP activity in neutrophil granules [70]. Other approaches to achieve fluorescent probes with fluorescence activation mechanisms or qABPs resemble those designed to image NE activity and have been reviewed elsewhere [32, 76].

### 4.2 MPO and HOCl

MPO is a nonproteolytic enzyme involved in NETosis. Therefore, a significant number of fluorescence probes used to detect MPO activity are indirect and instead focus on HOCl. While probes designed to sense HOCl are abundant and have been recently reviewed elsewhere [77], only a few have been used to detect MPO activity through HOCl in the context of neutrophil functions.

**FIGURE 4:**
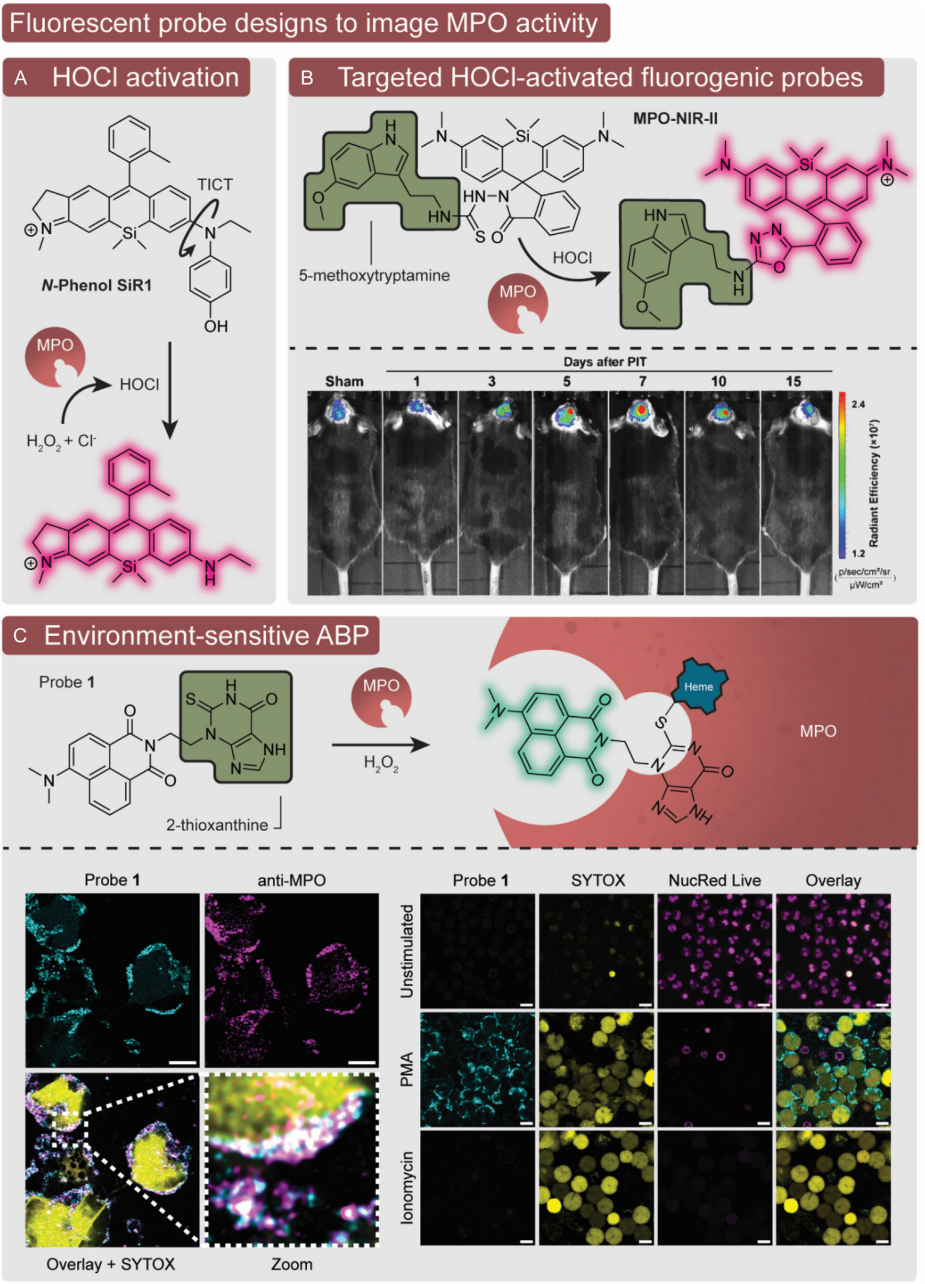
Overview of fluorescent probe designs to image MPO activity. (A) Caged fluorophores that rely on HOCl activation. (B) Targeted fluorogenic probes that rely on HOCl activation, exemplified by **MPO‐NIR‐II** (top). In vivo images from ischemic mice from 1 to 15 days post‐stroke after **MPO‐NIR‐II** injection (bottom). Reprinted (adapted) with permission from ref. [78]. Copyright 2025 American Chemical Society. (C) Environment‐sensitive ABP design (top) exemplified by probe **1**. Colocalization of probe **1** (cyan), anti‐MPO antibody (magenta), and NETs (SYTOX, yellow) in fixed neutrophils stimulated with PMA (bottom, left). Confocal microscopy panel of live neutrophils stimulated with PMA or ionomycin in the presence of probe **1** (cyan), SYTOX (NETs, yellow), and NucRed Live (DNA stain of live cells, magenta) (bottom, right). Scale bars = 10 µm. Reprinted (adapted) from ref. [16].

Most strategies are primarily based on fluorogenic substrates that use HOCl‐reactive caging groups (Figure 4A). Early work led to the development of a fluorescein derivative that, upon oxidation by HOCl, became highly fluorescent [79]. The probe was used in vitro with MPO to visualize HOCl production in PMA‐stimulated porcine neutrophils. Follow‐up research modified the probe towards longer emission wavelengths, and it was employed to image MPO activity in PMA‐stimulated human neutrophils and MPO^+^ macrophages, as well as HOCl generation in vivo and in human histopathological samples [80]. Since then, numerous fluorescent probes have been synthesized in an effort to improve sensitivity and applicability. More recent work by Ohno et al. reported a NIR probe for HOCl (***N*‐Phenol SiR1**) based on a Si‐rhodamine (SiR) fluorophore quenched by a twisted intramolecular charge transfer (TICT) mechanism [81]. The addition of a phenol ring to the SiR scaffold maintains the fluorophore in a TICT state, resulting in negligible fluorescence. Oxidation by HOCl removes the phenol ring, allowing the probe to become fluorescent and image MPO activity in HL‐60 cells, which naturally express MPO, NETosis stimulated by PMA, and phagocytosis of *E. coli* by murine neutrophils [81]. While in the context of NETosis HOCl production is exclusive to MPO, HOCl itself is highly reactive, very short‐lived, and diffuses rapidly. This can make the precise detection and spatiotemporal quantification of MPO activity challenging.

Some fluorescent probes for MPO use recognition elements that bind to the enzyme but still employ HOCl‐reactive caging groups (Figure 4B). For example, Liu et al. conjugated 4‐aminobenzoic acid hydrazide (ABAH), a known MPO inhibitor, to methylene blue (MB) to give a fluorescent probe able to bind MPO without inhibition [82]. The authors claimed that the probe did not inhibit MPO, which they attributed to steric hindrance by MB. The hydrazide bond connecting ABAH and MB can be cleaved by HOCl, thereby releasing MB and resulting in increased fluorescence. The probe was used to detect basal MPO activity levels in HL‐60 and MPO activity in vivo in arthritis mouse models [82]. Based on previous work, Qin et al. designed a NIR fluorogenic probe (**MPO‐NIR‐II**) by coupling a SiR‐thiosemicarbazide fluorophore to a 5‐methoxytryptamine moiety [78]. While in the native form, SiR‐thiosemicarbazide is nonfluorescent, and HOCl can promote its cyclization to a highly fluorescent SiR‐oxadiazole [83]. The probe was used to image HOCl production in vitro in HL‐60 cells and interferon‐γ‐stimulated murine neutrophils. MPO‐NIR‐II was also incorporated into a high‐content screening platform for MPO inhibitors using MPO‐overexpressing cells and ischemic brain slices. In addition, they investigated MPO activation and neutrophil infiltration ex vivo and in vivo in mice after ischemic stroke [78].

The first instance of an ABP targeted directly to image MPO activity was developed by Ward et al., who used a 2‐thioxanthine inhibitor to synthesize an alkyne‐tagged probe [84]. 2‐Thioxanthines have been reported as inhibitors of MPO, acting by irreversibly binding the catalytic heme group in an H_2_O_2_‐dependent mechanism [84, 85]. The probe was only used to detect MPO in vitro and in complex lysates in gel‐based analyses. Recently, our group conjugated a 2‐thioxanthine scaffold with an environment‐sensitive fluorophore, 4‐*N*,*N*‐dimethylamino‐1,8‐napthalimide (4‐DMN), to create a fluorogenic ABP for imaging MPO activity (probe **1**) (Figure 4C) [16]. The environmental change from an aqueous medium to the more hydrophobic enzyme‐probe binding site of MPO induces strong fluorescence. The probe was successfully applied in live‐cell imaging of NETosis and used to distinguish between PMA‐ and ionomycin‐induced NETosis, as MPO was only active in the former and not the latter.

### 4.3 MMPs

MMPs are Zn^2+^‐containing Ca^2+^‐dependent proteases characterized by their ability to degrade ECM proteins, intracellular proteins, and cytokines [86]. Of note are MMP‐8 and MMP‐9, which are stored in the tertiary granules of neutrophils. Specifically, studies have implicated MMP‐9 in pathologies associated with NETosis [87, 88]. A recent study found that wound healing in patients with type II diabetes may be impaired due to MMP‐9‐mediated collagen degradation during NETosis [87]. Others showed a positive correlation between MMP‐9‐abundant neutrophil subpopulations and increased NETosis in myocardial ischemia/reperfusion injuries, indicating increased severity and worse outcomes [88].

A variety of fluorescent imaging probes have been developed for MMPs, including MMP‐9, as reviewed elsewhere [89, 90]. However, they have not been employed to study neutrophil functions and NETosis. Possible explanations could be that MMP fluorogenic probes, such as FRET‐based peptides, suffer from poor selectivity due to conserved substrate recognition [90]. In addition, MMPs lack nucleophilic residues for covalent targeting, as they rely on Zn^2+^‐activated water molecules for catalysis, and approaches have instead relied on inhibitors coupled to photo‐crosslinkers [91].

**FIGURE 5:**
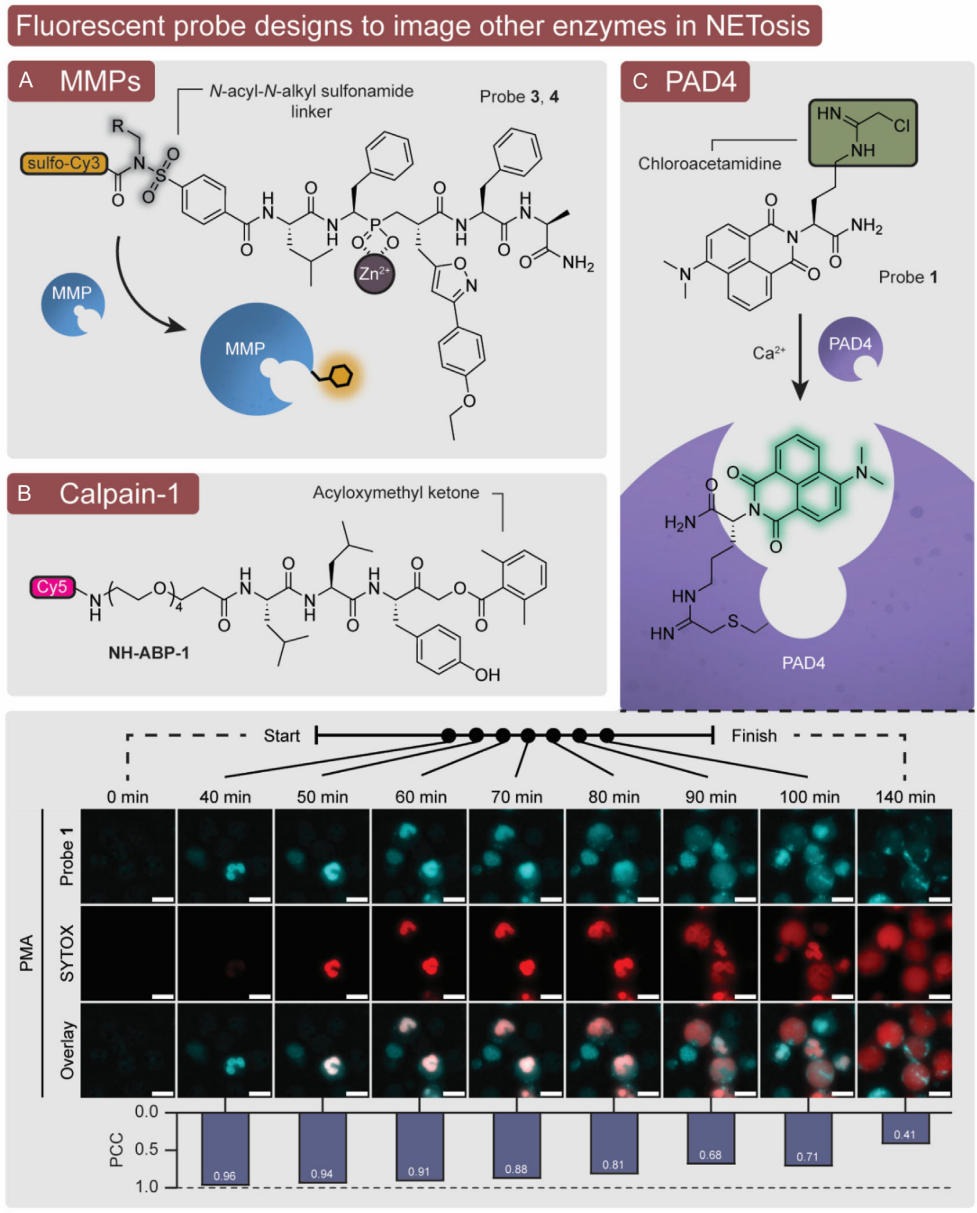
Overview of fluorescent probe designs to image the activity of other enzymes involved in NETosis. (A) AfBP to image MMPs. (B) ABP to image calpain‐1. (C) Environment‐sensitive ABP to image PAD4 (top). Confocal microscopy panel of a time‐lapse of live neutrophils stimulated with PMA in the presence of probe **1** (cyan) and SYTOX (NETs, red) (bottom). Scale bar = 10 µm. Reprinted (adapted) with permission from ref. [17]. Copyright 2026 American Chemical Society.

Recently, the Devel group introduced an affinity‐based probe (AfBP) containing phosphinic pseudo‐peptides bearing *N*‐acyl‐*N*‐alkyl sulfonamides that react selectively with a lysine in the S3 subsite of MMPs (Probe **3**, **4**) (Figure 5A) [92, 93]. This reaction results in the attachment of the tag to the enzyme and the subsequent release of the recognition element. While the probes have not been used to image MMP activity in the context of NETosis, they offer a promising new strategy for covalent MMP targeting.

### 4.4 Calpain‐1

Calpain‐1 is a Ca^2+^‐dependent cysteine protease that has been recently implicated in NETosis. It has been demonstrated that calpain‐1 cooperates with PAD4 to weaken the nuclear membrane and promote chromatin decondensation [26, 27]. While various inhibitors and fluorescent peptide‐based substrates have been developed to study calpain‐1, they generally lack selectivity and are prone to hydrolysis by related enzymes [94]. To address this, the Poręba group employed HyCoSuL (see above) to profile the substrate specificity of calpain‐1 and developed fluorogenic substrates, inhibitors, and the first calpain‐1–selective ABPs (**NH‐ABP‐1**) (Figure 5B) [95]. With these tools in hand, the authors visualized calpain‐1 activity in calcium ionophore A23187‐treated neutrophils, but not in resting or PMA‐treated neutrophils, using gel‐based analyses. In the future, these probes could be used for the real‐time live‐cell imaging of calpain‐1 activity during NETosis employing a FRET‐based approach.

### 4.5 PAD4

In a fashion similar to MPO, early efforts to image PAD4 activity focused on citrulline detection, often employing phenylglyoxal‐based probes [96, 97]. While these reagents enabled covalent modification and detection of citrullinated residues (i.e., detection of PAD4 activity), they required highly acidic conditions incompatible with physiological environments, thereby limiting their application in live‐cell imaging contexts. To overcome these challenges, the Thompson group developed ABPs for PAD4 based on mechanism‐based covalent inhibitors containing haloacetamidine electrophilic traps, such as Cl‐amidine and F‐amidine. These inhibitors were modified to generate alkyne‐functionalized derivatives that could be conjugated to fluorophore reporters. Their modular design enabled PAD4 activity detection in bacterial (*E. coli*) and mammalian (HL‐60‐derived granulocytes, MCF‐7) cells [98, 99, 100].

Inspired by these strategies, our group employed a similar strategy to design PAD4 ABPs that could be used to image PAD4 activity in live cells. We incorporated 4‐DMN into the Cl‐amidine scaffold to design an environment‐sensitive ABP capable of real‐time, wash‐free imaging of PAD4 activity, allowing the study of its activation dynamics during NETosis (probe **1**) (Figure 5C) [17]. Importantly, probe **1** was selective for PAD4 over PAD2, another PAD isoform expressed in neutrophils. Live‐cell imaging experiments further revealed that PAD4 was active in NADPH oxidase‐dependent and ‐independent NETosis pathways. However, its activation dynamics and subcellular localization varied with the stimulus, highlighting the context‐dependent regulation of this enzyme during neutrophil activation and suggesting a distinct mechanistic role in different NETosis programs. While the covalent nature of the probe was shown not to interfere with the dynamics of NETosis by itself, its blueshifted excitation spectra can interfere with the long‐term imaging of neutrophil functions, which have been shown to undergo NETosis upon exposure to blue and long‐wave UV irradiation [101].

Given the breadth of fluorescent probes developed to study neutrophil functions, we have selected key examples that have been specifically employed to image NETosis in live‐cell imaging settings and summarized them in Table 1. We state their molecular target, probe design, fluorescence activation mechanism, direct versus indirect reporting of enzymatic activity, applicability in primary neutrophils, and use in in vivo models, and key limitations. Beyond qualitative imaging, the probes discussed here can also be employed for quantitative measurements of enzymatic activity during NETosis. Ratiometric probes such as **H** **‐** **NE**, which rely on a FRET‐based design, are particularly well‐suited for this purpose, as the ratio of donor‐to‐acceptor emission provides a concentration‐independent readout of NE activity that can be correlated with the extent of NETosis across cell populations. Intensity‐based probes, including caged fluorophore designs and environment‐sensitive ABPs such as probe **1** for MPO and PAD4, allow fluorescence signals to be directly correlated with local enzymatic activity, enabling comparisons between different stimulation conditions or inhibitor treatments. Collectively, these features position the described probes not only as qualitative imaging tools, but also as quantitative reporters of the enzymatic landscape underlying NETosis.

**TABLE 1:** Comparative overview of fluorescent probes used to image NETosis in live‐cell imaging settings.

Probe name	Target	Probe design	Fluorescence activation	Direct vs. indirect	Used in primary neutrophils?	Used in vivo?	Key limitations	Reference
**H‐NE**	NE	Peptide with DNA‐targeting moiety	FRET pair (F + F)	Direct	Yes, human	Yes	Cell impermeable and reports NE activity on NETs only	[51]
Probe **4**	NE	qABP with electrophilic trap	FRET pair (F + Q)	Direct	Yes, human	No	Cell impermeable	[75]
***N*‐Phenol SiR1**	MPO	HOCl‐reactive SiR scaffold	Caging group, loss of TITC quenching	Indirect	Yes, murine	No	Signal diffusion	[81]
**MPO‐NIR‐II**	MPO	HOCl‐reactive SiR scaffold with 5‐methoxytryptamine	Fluorogenic substrate	Direct	Yes, murine	Yes	Limited human neutrophil NETosis data	[78]
Probe **1**	MPO	ABP with 2‐thioxanthine	Environment‐sensitive fluorophore	Direct	Yes, human	No	Covalent and blueshifted	[16]
Probe **1**	PAD4	ABP with chloroacetamidine	Environment‐sensitive fluorophore	Direct	Yes, human	No	Covalent and blueshifted	[17]

Abbreviations: F, fluorophore; Q, quencher.

## 5 Conclusions and Outlook

Fluorescent probes have become invaluable tools for studying neutrophil biology, as demonstrated by their wide range of applications. Among these, ABPs have provided particularly important advances by allowing the direct visualization of active enzymes rather than total protein abundance. This distinction has proven critical in neutrophils, where enzyme activation is highly regulated, transient, and often spatially restricted. One of the key insights gained from employing ABP‐based strategies is that enzyme activation during NETosis is not only stimulus‐dependent but also exhibits different temporal activation dynamics. For example, MPO‐targeted probes have revealed that its catalytic activity is tightly coupled to NADPH oxidase‐derived ROS production, and is largely absent in calcium ionophore‐induced NETosis. These findings have helped to clarify longstanding debates regarding the relative contributions of specific enzymes to NETosis pathways and highlight that enzymatic function cannot be inferred solely from protein localization or expression levels.

ABPs have also provided important insights into the subcellular localization of active enzymes. For instance, environment‐sensitive ABPs have shown differential PAD4 staining in NADPH oxidase‐dependent and ‐independent NETosis. During the early stages of NADPH oxidase‐dependent NETosis, PAD4 activation appears to precede plasma membrane rupture and highly colocalizes with decondensing chromatin. In contrast, PAD4 activation during NADPH oxidase‐independent NETosis seems to be a late event that occurs after plasma membrane rupture. Real‐time imaging of PAD4 is beginning to reveal how its activation correlates with calcium flux and chromatin decondensation, offering a more detailed understanding of how the molecular mechanisms of NETosis are orchestrated in space and time.

Despite these advances, several challenges remain. Many current probes are peptide‐based: while they often provide high selectivity over related family members, their size and hydrophilicity limit plasma membrane permeability and restrict their utility for live‐cell imaging, which is an essential requirement for capturing the rapid and highly dynamic neutrophil effector functions. Alternative design strategies, such as caged fluorophores, have circumvented some limitations for NE, but similar approaches remain underdeveloped for other NSPs, MMPs, and calpain‐1. At the same time, the emphasis on proteases has left nonproteolytic enzymes underexplored.

Addressing these challenges will require the generation of improved recognition elements with greater specificity, broader chemical diversity, and compatibility with physiologically relevant environments. Expanding substrate profiling platforms and covalent inhibitor discovery could yield the next generation of fluorescent probes for selective and minimally perturbative labeling. Moreover, the development of smaller, more cell‐permeable probes and optimized fluorescence activation strategies, such as self‐immolative linkers, qABPs, and new environment‐sensitive fluorophores, will be essential for enhancing signal‐to‐background ratios and enabling real‐time imaging without washing steps, a particular need during NETosis. Future advancements will also benefit from designing probes that can function in more complex systems. NETosis is highly context‐dependent and influenced by factors that are not fully represented by stimuli like PMA or calcium ionophores. Thus, probes optimized for primary human samples, ex vivo tissues, and intravital imaging will be critical to investigate how NETosis unfolds under physiologically relevant conditions.

Together, the continued development of fluorescent probes that overcome these limitations will enable the study of neutrophil responses with greater spatial and temporal resolution, deepening our mechanistic understanding of NETosis and other neutrophil effector functions. Beyond descriptive imaging, next‐generation probes hold the potential to uncover pathogenic neutrophil states across inflammatory diseases, autoimmunity, cancer, and neurodegeneration. Ultimately, these chemical tools will not only enhance fundamental insights into neutrophil biology but also facilitate translational efforts aimed at therapeutically modulating neutrophil activity in disease.

## Funding

This work was supported by H2020 European Research Council (Grant 802940), Nederlandse Organisatie voor Wetenschappelijk Onderzoek (Grant NWO‐024.002.009).

## Conflicts of Interest

The authors declare no conflicts of interest.

## Data Availability

The data that support the findings of this study are available from the corresponding author upon reasonable request.
